# Barriers and enablers to walking in individuals with intermittent claudication: A systematic review to conceptualize a relevant and patient-centered program

**DOI:** 10.1371/journal.pone.0201095

**Published:** 2018-07-26

**Authors:** Ukachukwu Abaraogu, Elochukwu Ezenwankwo, Philippa Dall, Garry Tew, Wesley Stuart, Julie Brittenden, Chris Seenan

**Affiliations:** 1 University of Nigeria Department of Medical Rehabilitation, Enugu, Nigeria; 2 Glasgow Caledonian University School of Health and Life Sciences, Glasgow, United Kingdom; 3 Northumbria University Department of Sport, Exercise and Rehabilitation, Newcastle, United Kingdom; 4 Vascular Surgery NHS Greater Glasgow and Clyde Health Board, Glasgow, United Kingdom; 5 Institute of Cardiovascular and Medical Sciences University of Glasgow, Glasgow, United Kingdom; University of Hull, UNITED KINGDOM

## Abstract

**Background:**

Walking limitation in patients with peripheral arterial disease (PAD) and intermittent claudication (IC) contributes to poorer disease outcomes. Identifying and examining barriers to walking may be an important step in developing a comprehensive patient-centered self-management intervention to promote walking in this population.

**Aim:**

To systematically review the literature regarding barriers and enablers to walking exercise in individuals with IC.

**Methods:**

A systematic review was conducted utilizing integrative review methodology. Five electronic databases and the reference lists of relevant studies were searched. Findings were categorized into personal, walking activity related, and environmental barriers and enablers using a social cognitive framework.

**Results:**

Eighteen studies including quantitative (n = 12), qualitative (n = 5), and mixed method (n = 1) designs, and reporting data from a total of 4376 patients with IC, were included in the review. The most frequently reported barriers to engaging in walking were comorbid health concerns, walking induced pain, lack of knowledge (e.g. about the disease pathology and walking recommendations), and poor walking capacity. The most frequently reported enablers were cognitive coping strategies, good support systems, and receiving specific instructions to walk. Findings suggest additionally that wider behavioral and environmental obstacles should be addressed in a patient-centered self-management intervention.

**Conclusions:**

This review has identified multidimensional factors influencing walking in patients with IC. Within the social cognitive framework, these factors fall within patient level factors (e.g. comorbid health concerns), walking related factors (e.g. claudication pain), and environmental factors (e.g. support systems). These factors are worth considering when developing self-management interventions to increase walking in patients with IC. Systematic review registration CRD42018070418.

## Introduction

Intermittent claudication(IC), defined as exertional leg pain which goes away with rest, is a common symptom in patients with peripheral arterial disease (PAD). IC in patients with PAD reduces walking capacity, quality of life and is associated with increased cardiovascular risk[1,2]. Individuals with IC have less than half the maximal walking capacity [3], and up to 3–4 time increased risk of mortality compared with their age matched control[4]. Management of the condition aims to improve longer-term cardiovascular outcomes through risk factor modification while reducing claudication symptoms, with conservative management being the first to be considered[5,6]. Walking exercise is the most effective conservative management for reducing leg symptoms[7], and a supervised exercise program (SEP)[5,8–10] or a home-based exercise program (HEP) is recommended[11].The rationale for recommending walking exercise in IC includes both the benefit of symptom relief and cardiovascular risk management[10,12–14]. For patients with IC symptoms to gain these benefits, walking beyond the point of pain is recommended[5][11], representing a potential barrier to uptake and adherence to the therapeutic recommendation.

Efforts to engage individuals with IC in therapeutic walking exercise have been challenging on several fronts. For instance, SEPs for patients with IC experience difficulty with patient engagement and high levels of attrition[15]. Even when patients successfully complete a SEP, there are barriers of translating and sustaining the walking ability gained in the clinic to walking behavior in the free-living environment[16,17]. Similarly, demonstrating the benefits and economic viability of home-based walking exercise in patients with PAD and IC has been challenging due to patient attrition, non-adherence and other issues related to barriers to home-based walking exercise[18]. McDermott et al reported that factors related to walking in patients with PAD go beyond the barrier of claudication pain[19], underscoring that complex multi-level factors influence whether or not patients take up, and adhere to, therapeutic walking exercise recommendations. To improve the participation of individuals with IC in walking exercise, it is important to understand the reasons why they do not engage in walking exercise and the enablers that could be useful to develop walking intervention in this population. This supports the growing interest in self-management approach in treating patients with IC.

Within the conceptual framework of self-management interventions, evidence indicates that the inclusion of behavior-change techniques contribute to the improvement of pain free walking ability, self-reported walking ability and daily walking activity in individuals with IC[20–22]. Several important behavioral change techniques such as barrier identification with problem-solving[20,23], self-monitoring[21,23], feedback on performance[21,23], goal setting[20,23], social support[23], action planning[20], and structured patients education[24] have been highlighted. However, to develop targeted evidence-based self-management interventions informed by behavioral change strategies, a clear understating of the multifactorial barriers and enablers common to IC patients’ participation in walking exercise is required. Identification of these factors via a systematic review, perhaps using a conceptual framework, may be the first step to guide the development of a suitable patient-centered walking intervention.

One previous review[25] has been conducted to provide generic understanding of barriers and facilitators to walking in PAD. PAD is an overarching construct and includes a range of symptom severity from asymptomatic to severe critical limb ischemia with ulceration and gangrene. Therefore, barriers to walking may be unique to each stage of the disease. However, the previous review included patients at different stages of the disease pathway making it impossible to understand what factors were specific to patients with IC. Similarly, only literature published between 2010 and 2016 was included in the previous review. Finally, the previous review conclusion was limited to older adults (≥65years) with PAD. Although age is a significant risk factor in PAD, young adults are increasingly affected[26,27]. Moreover, age-mediated variations in the clinical symptomology of PAD suggest that claudication is more prevalent in the younger patients (≤50years)[28]. The aim of this review is to systematically review the literature regarding both self-report and objective measures of barriers and enablers to walking exercise in individuals with IC.

## Materials and methods

**Fig 1:**
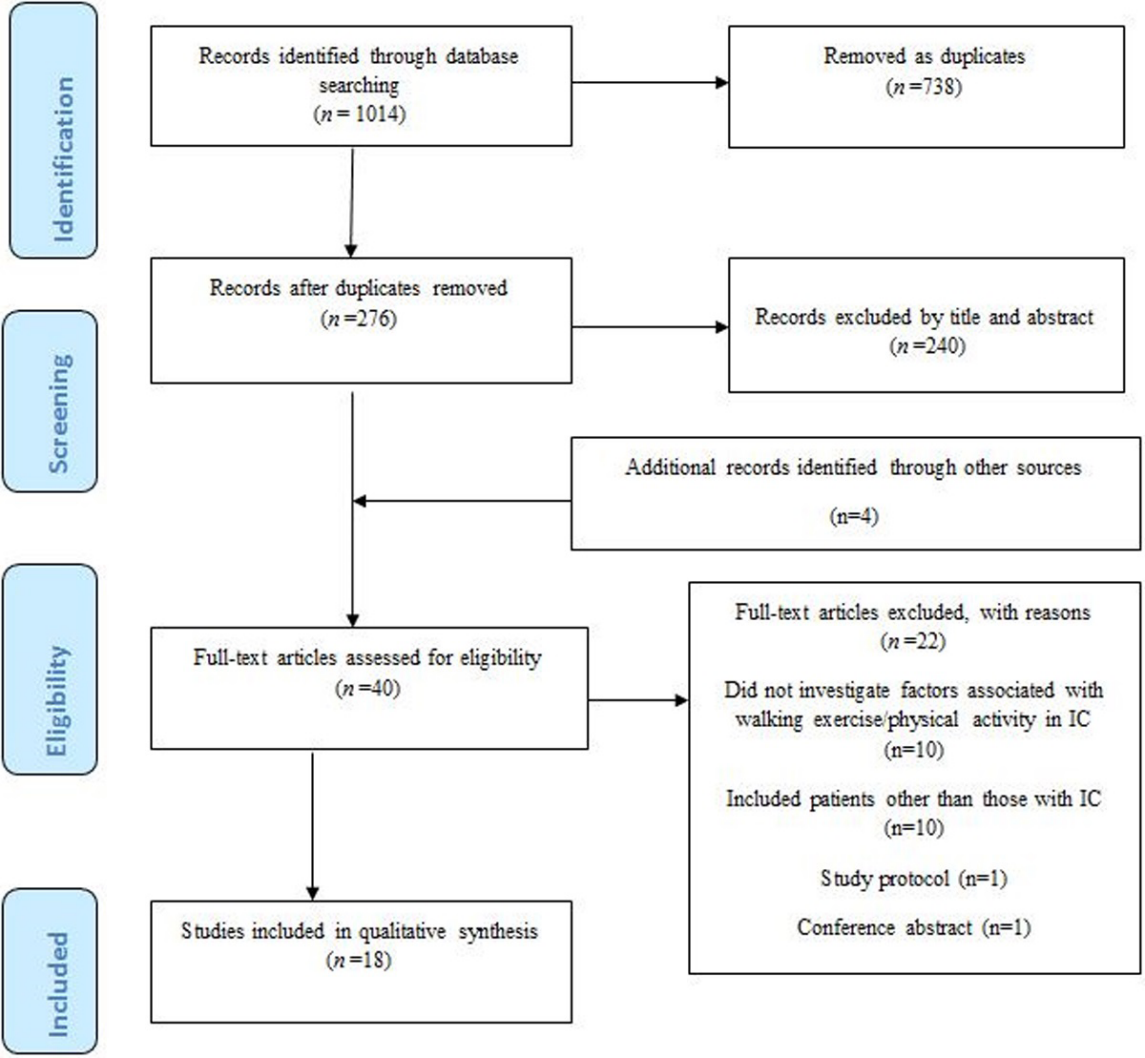
PRISMA flow diagram for systematic review of barriers and enablers to walking in individuals with intermittent claudication.

Preferred Reporting Items for Systematic Reviews and Meta-Analyses (PRISMA) (where applicable) and the four-phase item flow diagram guidelines were followed[29] (See Fig 1). The review protocol was registered with PROSPERO (CRD42018070418).

### Design

A systematic review was planned to explore two research questions: 1) what is the perception and provision of routine supervised exercised therapy for PAD, and 2) what are the barriers and enablers to walking in IC? Given, that the two proposed review questions are tangential to each other, requiring different search strategies and inclusion criteria, the decision was made to execute the two research questions in separate reviews for clarity and ease of synthesizing the findings.

This present paper reports on the barriers and enablers of walking exercise in patients with IC, and was conducted using the integrative review strategy reported by Whitmore and Knafl [30]. An integrative review is a specific review approach that summarizes evidence from research of diverse methodologies (empirical or theoretical literature), and may be used to frame a more comprehensive understanding of a particular phenomenon or healthcare problem[31], such as factors influencing IC patients engagement in therapeutic walking. Integrative review methodology was selected as it is the only approach that would enable a systematic evidence synthesis from both quantitative and qualitative data. Inclusion of both types of data is desirable in this review to gain a comprehensive understanding of the review topic, and will be useful to begin to build knowledge about the concept of walking intervention in this population. The overarching aim of the review is to utilize the understanding of the barriers and enablers to walking in patients with PAD and IC to conceptualize a patient-centered walking program.

This integrative review entailed a systematic literature search, data extraction, quality appraisal, and data synthesis via a deductive framework aggregation of findings into conceptual units using the Social Cognitive Theory Framework[32]. First proposed by Bandura[32,33], social cognitive theory explains how people acquire and maintain certain behavioral patterns, while also providing the basis for intervention strategies[34]. The use of social cognitive theory has been increasingly identified as a viable framework to implement a review to guide intervention development for physical activity in chronic conditions[35–37], such as IC. This review was framed using the social cognitive perspective, as it allowed the examination of the multiple levels of factors and their influence in walking among patients with IC, providing insight into important considerations for developing walking interventions.

### Study eligibility criteria

#### Types of participants

Studies reporting on adults (≥18years) with PAD and IC were eligible. To be included, all study participants must have been specifically diagnosed with IC. Diagnosis could have been by having Fontaine stage II PAD, or its equivalents, or if it was stated that only patients with IC were included in the study. Studies with participants having critical limb ischemia (rest leg pain, ischemic tissue loss, or an ABI<0.4) were excluded.

#### Types of studies

Quantitative, qualitative and mixed methods studies in English language literature were eligible if published in peer review journals, or conference proceedings, and reporting primary data on barriers, enablers, facilitators or motivators for walking exercise and/or physical activity in IC. In addition to studies which examined the barriers or enablers for walking (either cross-sectional or longitudinal), studies reporting on experiences of living with IC were included if they specifically mentioned barriers or motivation or facilitators to walking. Also eligible were intervention studies that reported on factors influencing walking outcomes.

#### Context

Studies with any cultural, healthcare, geographical, or community contexts were included.

### Identification of primary research studies

A systematic literature search was implemented until January 2018 (updated in June 2018) in five databases (CINAHL via EBSCO, MEDLINE via ProQuest, AMED via Ovid, Science direct, Social citation index/ Science citation index /Emerging sources citation index via Web of Science) by the first author (UA). The following key words, and medical headings in combination with database specific search syntax, filters, limiters, and Boolean operators were used: Peripheral arterial disease OR Peripheral vascular disease OR Peripheral occlusive disease OR Intermittent claudication OR Claudication pain AND Quality of life, OR Patient reported experience OR Patient experience OR Illness beliefs OR Factors OR Enablers OR Motivators OR Barriers OR Facilitators AND Walking OR Walking exercise OR Supervised exercise OR Physical activity OR Exercise. Further searches were made to identify studies from the reference lists of relevant studies. A sample search strategy is presented in the supporting information.

#### Data management

Studies were exported to remove the duplicates in Refworks™ and then exported to Microsoft Excel. Two review authors (UA, EE) read the title, and abstract, followed by full text to identify eligible studies against the previously defined eligibility criteria. Studies without a self-report or objective finding on factors related to walking, or studies without a homogenous sample of individuals with IC were excluded. In cases of divergence in analysis of eligibility decision, studies were discussed until a consensus was reached, or a third author (CS) was consulted.

### Data extraction processes

The data extraction was independently conducted by two review authors (UA, EE), with the results discussed afterwards to reach a consensus. A customized data extraction form specifically developed and piloted for this review was utilized. Data forms included author’s details (author, year, and country), study aim, study design, level of evidence, sample characteristics/cultural context, variables, results, authors’ main conclusion. When an article was only available as an abstract email requests for the full length of details of an article were made (up to twice) to the study author. The article was excluded if there was no response or if the full detail was not available. The table of characteristics of included studies is presented in Table 1.

**Table 1:** Table of data extraction and characteristics of included studies.

Author details, date, country	Study aim(s)	Study design, Analysis, Level of evidence	Sample and context	Variables	Authors main conclusions
Bartelink et al.[38] Netherlands	Report factors which affected walking behavior in patients with IC	MM Regressions analysis LoE: VI	-Cross sectional study n = 216; 69%Male -Focus group n = 9 -Dutch primary care patients. -Age: 66.9y (range42-97yrs; -≤Secondary education (85%); Pulmonary disease (50%); Osteoarthritis (47%); Hypertension(33); Hypercholesterolemia (30%); Myocardial infarction (20%); Angina pectoris (22%); Diabetes (17%); Minor stroke/stroke (9%)	IV: Facilitators & barriers to walking exercise DV: Walking exercise	Lack of advice, unspecific advice, & lack of supervision were important barriers for performing walking exercise.
Galea et al.[39] Canada	Identify barriers & facilitators associated with walking exercise in patients with IC	Focus group interview Content analysis LoE: VI	N = 15 Diagnosis of IC & ABI<0.9 47%Male Age (mean = 76.9, range 54-89y) Mean ABI = 0.67	NA	Barriers to walking in patients with IC included irregular surfaces, uncertainty about walking, pain, & need to rest; Enablers are availability of resting place, cognitive strategy, support & SEP availability.
Galea et al. [40] United Kingdom	Explore patients experiences of & belief about illness & walking with IC	Semi-structured interviews FA LoE: VI	n = 19 Age: Mean 66 (range 44–79)y 13 Male Longstanding IC(≥2y): 53%	NA	Illness & treatment uncertainties may explain the low participation in walking in patients with IC
Gorely et al.[41] United Kingdom	Explore experiences of IC & thoughts on walking as an intervention	Focus group TA LoE: VI	N = 24; 71%Male; White British; Age: mean (71y); Duration of IC: median 17.5, range 3-18months.	NA	Addressing the knowledge gap & uncertainties around the disease process & walking is needed enhance behavior in patients with IC.
Cavalcante et al.[42] Brazil	Investigate sociodemographic commodities & clinical variables barriers to PA	Cross-sectional Regressions analysis LoE: VI	N = 145; Sociodemographic: Age(≥65y, 55%); 65%Male; Race(39%, Non-white); Married status(67%, Married or living with a partner); Economic level(36% Low income) Comorbidities: Hypertension (81%); Dyslipidemia (72%); Overweight (60%); Cardiac disease (59%); Diabetes(41%); Current smoker (24%)	IV: Demographic variables; Comorbid conditions DV: ABI, ICD, ACD.	Patients with IC who are older, with lower economic status, diabetes, low ABI and walking capacity are more likely to experience barrier to physical activity.
Harwood et al.[43] United Kingdom	Understand perceptions including barriers & facilitators, to SEPs	Qualitative interview TA LoE: VI	Patients who declined, withdrew from, or completed supervised exercise program. N = 25; 56%Male; Age: mean = 71, range = 44–79	NA	More education or time investment is required at initial diagnosis to overcome patients’ barriers to healthy behavioral changes.
Sharath et al.[44] USA	Examine relationship between pain belief & (each of) symptom severity, expectation, & baseline PA	Cross-sectional Quantitative analysis LoE: VI	N = 20; Age: mean69y, IQR 66–75; BMI: median 28[IQR, 20–30]; 95%Male; Ethnicity: Caucasian (55%), African American (40%); High school education (100%) ABI: median 0.6; *Comorbidities*: COPD (25%), Hypertension (80%), Hyperlipidemia (60%), Diabetes (20%).	IV: Pain belief & perceptions using the Fear-Avoidance Belief Questionnaire DV: Daily PA	Engaging in walking in patients with IC is positively related to symptoms severity & underscore the importance of considering patients belief about pain in interventions to increase walking
Barbosa et al.[45] Brazil	Analyze factors associated with PA	Cross-sectional Regression LoE: VI	N = 150; Age = 64±9; 63%Male; BMI = 26±4.5; ABI = 0.59±1.54; Duration since diagnosis ≤8y = 72%; Low income = 35%; Diabetes = 43%; Hyperlipidemia = 92%; Dyslipidemia = 87%; Cardiac disease = 56%; Currently smoking = 23%	IV: Barriers to walking DV: PA	Older adults in neighborhoods without access to green areas for walking, & who present poor walking capacity have lower PA.
Egberg et al.[46] Sweden	Describe experiences of patients about living with IC	Qualitative interview TA LoE: VI	N = 15; 47%Female; Age = mean(73y), range(64-81y)	NA	Experience of living with IC depends on how active a patient is or wants to be, & underscores the need to understand this experience in treating IC
Farah et al.[47] Brazil	Predicting walking capacity using clinical characteristics & WIQ	Quantitative non-experimental LoE: IV	N = 133; 64.7%Male; Age (mean, 63±8.8; range, 30-80y); BMI = 26.4±4.6; ABI = 0.59±0.15; Smoking history = 84.2%; Hypertension = 76.7%; Dyslipidemia = 70.7%; Diabetes = 38.3%; Coronary artery disease = 56.4%.	IV: Demographic & clinical characteristics DV: ICD, ACD	It is feasible to estimate walking capacity in patients with IC using clinical characteristics & WIQ.
Dörentamp at al.[48] Netherland	Assess associations of demographic & clinical variables during & after SEP	Prospective cohort Regression analysis LoE: IV	N = 2995; 1864Male; Dutch patients with IC attending community-based SET and who have ICD <1600m at baseline; Age (mean = 67y); Vascular comorbidity 62%; Internal comorbidity (54%); Cardiac comorbidity (49%)	IV: Age, gender, BMI DV: ICD	Being female, advanced age, higher BMI, & having a cardiac comorbidity are associated with less improvement in ICD ability after SET in IC patients.
Gardner et al.[49] USA	Compare gender variations baseline clinical variable, & changes in ambulatory outcomes due to exercise training	RCT Regression analysis LoE: II	N = 48; Patient characteristics *(M*,*F)*: ABI(0.66, 0.69) BMI(28.7, 30.1); Caucasian race(65%, 40%); Smokers (30%, 48); Hypertension(835, 92%), Dyslipidemia(74%, 76%), Diabetes(355, 64%), Obesity(39%, 52%); Metabolic syndrome(78%, 88%)), Abdominal obesity(39%, 64%); Lower extremity revascularization(26%, 35%); Previous history of angina(265, 24%); Cerebral vascular accident(175, 12%); COPD(30%, 36%)	IV: Gender; DV: Exercise measure & ambulatory outcomes e.g. ICD, ACD	As women showed less improvement in peak walking distance in an onsite supervised exercise program, obese men and patients with low claudication onset time were least responsive to the program
Gardner et al.[50] USA	Determine if baseline variable, AND dose of ambulation during a HBE program predict ambulatory outcomes	RCT Regression analysis LoE: II	N = 46; 22Male; Mean Personal characteristics: (Age(66, 68y); ABI (0.71, 0.66); BMI(29.4, 28.3) *Demographic/ Clinical characteristics(M*,*F)*, *%*: Caucasian (59, 63); Smoking (23, 42); Hypertension (86, 92); Dyslipidemia (82, 88); Diabetes (36, 46); Obesity(41, 41); Abdominal obesity (55, 58); Metabolic syndrome (82, 92); Revascularization (27, 50); Angina (27, 21); CVA (18, 33); COPD (14, 33)	IV: Exercise cadence & time, Age, Smoking, ABI, Race, Metabolic syndrome, COPD, Revascularization DV: COT, PWT	While faster ambulatory cadence may predict greater improvement in ambulatory function in women with IC, less severity and lower comorbid burden are the predictors in men.
Fritsch et al.[51] USA	To investigate the effect of smoking on walking ability	Cross-sectional Descriptive & t-test LoE: VI	N = 105; Age: 70±9.1y; 92%Male; Current smokers: 34%; Race: 80%Caucacians; Heart disease: 31%; Diabetes: 65%;	IV: Current smoking status DV: ICD, MWD	PAD patients who smoke have lower ICD compared to those who do not.
Kruidenier et al[52] Netherland	To identify predictors of walking distance following a SEP	Prospective non-experimental Mann-Whitney *U* & χ^2^ analysis LoE: VI	N = 129; Male:88; Age:65.6±9.9; BMI:26.5±4.4; Resting ABI:0.71±0.21; SBP: 156.7±26.0; Current smokers:42%; Hypertension: 78%; Diabetes: 28.%; Pulmonary disease: 17%; Neurological disease: 52%; Cardiac disease: 34%; Orthopedic disease: 12%	IV: Clinical characteristics &baseline ACD DV: post treatment ACD & % change in ACD	Baseline ACD, BMI, and current smoking status are predictive of the value of ACD post-treatment with SET.
Galea et al.[53] Canada	To identify psychosocial determinants of walking exercise and the mediating role of pain in the intention-behavior gap	Prospective non-experimental Descriptive, Correlation & Regression analyses LoE: IV	N = 94; 65%Male; Age = 70.05±9.02; Ethnicity: White = 94.7%; Marital status: Married = 65%; Education level: ≥Secondary = 61%; Smoking status: Currently smoker = 34%; Treadmill exercise program participation: Currently enrolled = 37%; Disease location: Unilateral = 59%; Claudication symptom duration: >2y = 64%; Pharmacological pain treatment: Yes = 15%.	IV: Attitude & perception of walking; Perceived behavior control; Walking intentions; Pain intensity. DV: Walking exercise	While pain cognitions do not influence walking in patients with IC, the theory of planned behavior may be used to predict walking intentions and exercise in this patient population
Aherne et al.[54] Ireland	Investigate patients’ exercise participation & compliance & factors influencing patients outcomes	Prospective Observational cohort Descriptive & Regression analysis LoE: IV	N = 98; 82%Male; Age(mean = 69.2±10.1) Education: 39% had ≥Secondary education Current smokers: 37% Chronic obstructive pulmonary disease: 14% Ischemic heart disease: 20% Chronic kidney disease: 7% Diabetes: 20% Hypertension: 61% Hypercholesterolemia: 78%	IV: NA DV: Total number of exercise session a patient attended	Improvement in function of SEP and patients’ compliance may be gained by pre-exercise patients’ education and personalized exercise prescription.
Cornelis et al.[55]	Identify barriers to PA & needs & interest for technology-based exercise	Cross-sectional Descriptive & correlation LoE: IV	N = 99; 76Male; Mean age: 69y; 81% Retired; 65% with at least a secondary education; 53% had bilateral symptoms; 28% Smokers; 92% Hyperlipidaemia; 92% Hypertension; 30% Diabetes mellitus;	IV: Barriers to PA DV: PA levels	Pain & obstacles worsening pain are the major barriers to PA in IC.

Key: CA: Content analysis; FA: Framework analysis; IV: Independent variable; DV: Dependent variable; NA: Not applicable; SEP: Supervised exercise program; IHD: Ischemic heart disease; ICD: Initial claudication distance; ACD: absolute claudication distance; CI: Confidence interval; HBE: Homebased exercise; LoE: Level of evidence; COPD: Chronic obstructive pulmonary disease; TA: Thematic analysis.

### Critical appraisal/level of evidence

The methodological quality of the included studies was accessed using the Critical Appraisal Skills Program (CASP) instrument[56]. Specifically, the CASP Qualitative Checklist, CASP Randomised Controlled Trial Checklist, or CASP Cohort Study Checklist was selected as appropriate to appraise studies. CASP is a generic quality appraisal tool providing guidelines for appraising studies of range of methodological designs, and consists of nine to twelve questions which are answered with ‘yes’, ‘no’, or ‘can’t tell’. The initial two questions in the CASP checklist are screening questions related to the study aim and methodology, and have to be positively answered for the study to meet quality criterial to continue in the evaluation and review. If a study merits inclusion following a “yes” response to the first two CASP questions, the level of evidence in the study was subsequently assigned following the Melnyk and Fineout-Overholt guidelines[57]. In summary, a study with the highest and most robust level of evidence (systematic reviews) is assigned Level I, whereas the lowest evidence (from the expert opinion of authorities and/or reports of expert committees) is given Level VII. Discrepancies in ratings between authors were resolved by discussions in consultation with the review team.

### Data synthesis

Using the social-cognitive framework[32,33], a deductive framework synthesis approach was implemented to aggregate findings within conceptual units. We did not set a prior list of what constituted barriers or enables, but identified these as they were reported in the included studies. These barriers and enablers were then classified into those related to the individual (personal), to walking activity (behavioral) or to the environment (environmental). This synthesis method fitted within the review objective as it provided a highly structured and detailed approach to analyzing and organizing data from included studies. Two authors (UA, EE) independently undertook coding of findings, and mapping of barriers and enablers to the framework. The synthesis decisions were reviewed by the authors until consensus was reached.

## Results

### Study selection

The process of identifying, screening, and studies inclusion is summarized in a PRISMA flow chart (See Fig 1).

### Characteristics of included studies

Eighteen studies, consisting of quantitative (n = 12)[42,44,45,47–54], qualitative (n = 5)[39–41,43,46] and mixed method (n = 1)[38] designs, and involving a total of 3023 participants with IC, were included in the final review. Study participants ranged from 15[39][46] to 1741[48]. Included studies were from a total of eight countries with most from the USA (n = 4), followed by Brazil (n = 3), Netherland (n = 3), United Kingdom (n = 3), Canada (n = 2), Republic of Ireland (n = 1), Belgium (n = 1), and Sweden (n = 1)(Table 1). Most of the included studies(n = 10)[38,39,43,46–48,50,52,54,55] did not report data on the ethnic background of participants. Where reported, all studies (n = 8) had a majority of white participants[40–42,44,49–51,53]. Participants’ educational background was not reported in the majority (n = 13) of the included studies [39,40,43,44,46–52,58]. Where reported, most of the participants had completed at least either primary[38,42,54], or secondary[53][55] education. Only one study reported participants’ income status with most participants of high income[42]. Only two studies had data on participants’ marital status with the majority of the participants married or living with a partner[42,53]. Most (2815; 64%) of the participants in the included studies were male. Participants’ age in included studies ranged between 30–97 years. Although the disease duration, severity and comorbid burden varied between studies, all the included studies comprised only participants with a confirmed diagnosis of symptomatic PAD defined as IC. Where reported, the mean/median resting ABI reported ranged from 0.59[42,45,47,52] to 0.71[50,52], while disease duration since diagnosis ranged from newly diagnosed [40,53] to over two years[40,53].

### Quality of evidence/level of evidence

All eighteen studies were rated based on the CASP criteria. On the Melnyk and Fine-Overholt evidence hierarchy [57] evidence level in included studies ranged Level VI (n = 11), Level IV (n = 5), to Level II (n = 2). Although the majority of the studies were qualitative and non-experimental and the level of level of evidence was mostly moderate to low, these types of studies fitted within the review objective, and were included in the review.

### Barriers to walking exercise

**Fig 2:**
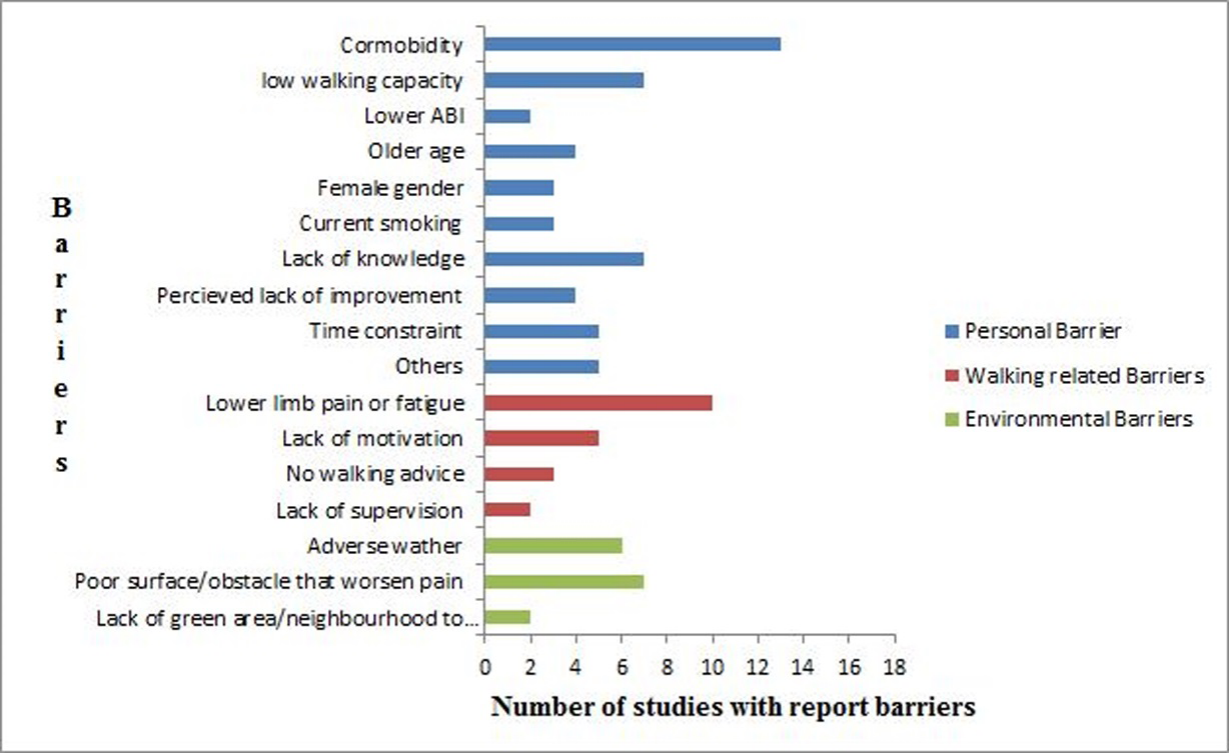
Barriers to walking in individuals with intermittent claudication and number of studies which reported them.

The number of studies reporting on different dimensions of barriers to walking is illustrated in Fig 2.

#### Person related barriers

Comorbid health factors: Comorbid health concerns were the most frequently identified barriers among participants. These barriers were reported by four (80%) of the qualitative studies[39–41,43] and were examined by nine (75%) of the quantitative studies[42,44,45,47–50,52,55] and the mixed method study[38]. Participants across the qualitative and mixed methods studies cited preexisting medical conditions and comorbidity as barrier to engaging in walking exercise[38–41], or dropping-out from a SEP program[43].The nine quantitative studies examined baseline factors associated with engaging in walking exercise and physical activity[42,45,47] or predictors of walking outcomes following SEP intervention[48–50,52]. Barbosa et al. reported that comorbid health conditions were a prevalent barrier to physical activity in 77 (51%) of their participants[45]. Diabetes was associated with decreased physical activity[45], lack of physical energy, personal barriers to physical activity[42], and was a predictor of poorer pain-free and maximum walking distances[47]. Similarly, higher BMI independently predicted lower improvement in pain-free[48] and maximum walking distance[52], and obesity predicted lower physical activity levels[45], and poorer improvement in both pain-free and maximum walking distance in men with IC[49]. Other comorbid conditions reported as barriers to physical activity included hypertension, arthritis, angina[45], and metabolic syndrome[45][50]. Participants across several studies also reported avoiding walking exercise or physical activity due to fear of falling, fatigue, injury or other adverse health issues[40–42,44,45,55].

Low walking capacity (walking capacity is the distance or length of time patients with IC can walk before pain symptoms are felt, or the pain experienced causes them to stop walking): Participants in one qualitative study reported that low walking capacity prevented them from moving around[46]. Seven quantitative studies examined the association between participants’ walking capacity measures and physical activity, or the role of participants’ walking capacity in mediating walking outcomes following SEP. These studies all indicated that low walking capacity was associated with lower physical activity[42,45,47], and lower pain-free and maximum walking distances following SEP, especially in women[49,50,52]. Also a lack of physical energy or inability to exercise at appropriate level was associated with decreased in walking exercise engagement[45][54].

Lower ABI: Two quantitative studies reported lower ABI as a barrier. Low ABI was associated with “some difficulty in getting to a place where physical activity can be performed”[42]. Similarly, poorer ABI predicted poorer improvement in 6-minutes walking distance of participants after a home-based exercise program[50]

Age: Participants in one qualitative study cited age as a barrier[41]. Three quantitative studies reported older age (>65 years) was a barrier to more physical activity engagement[42], and was associated with lower physical activity[45], or poorer improvement in pain free walking distance after SEPs[48].

Female gender: Three quantitative studies examined the influence of gender in ambulatory outcomes during and/ or after SEP. These studies showed that being female was associated with relative poorer improvement in pain free walking distance[48,49]. Also a lower mean exercise cadence predicted poorer pain free and maximal walking distances only in female participants[50].

Current smoking history: Three quantitative studies examined current smoking history as factors influencing walking ability or walking exercise engagement. Current smokers had lower pain-free walking distance than non-smokers[51]. Current smoking also predicted lower maximal walking distance following an exercise program[52]. Finally, current smokers had a lower mean attendance at exercise sessions compared with non-active smokers[54].

Lack of knowledge: Three major themes relating to lack of knowledge as a barrier to walking exercise were identified by the qualitative studies. The first theme was that participants did not engage in walking exercise because they lacked understanding of the pathology of IC[40,41]. The second theme was the lack of understanding and uncertainty regarding walking guidelines or suitable therapeutic walking dosages[41]. The third theme was that participants did not understand, or were uncertain, regarding the benefit of walking exercises, and how risk factors work[39–41,45,55]. Similar to this was participants’ outright disregard of walking as a treatment for IC[40]: “There’s no treatment. I’m getting no treatment, not for this. I’m getting advice, and the advice is ‘try to walk through it’. That’s the only advice I’ve ever had”[40]. Incidentally, the quantitative studies found that lack of knowledge was associated with low education level[42], and also that it predicted walking intention[53].

Perceived lack of improvement: One mixed methods study[38] and two qualitative studies[40,41] reported perceived lack of symptom improvement or lack of confidence that walking was providing any benefit as a barrier to walking exercise. Also, one quantitative study reported that subjective feelings of supervised training not providing any benefit was a barrier to participants continued attendance at SEPs[54].

Lack of time: The barrier of lack of time was identified in two quantities and three qualitative studies. Reasons for lack of time included associated burden of a hospital-based SEP[40,43], having other responsibilities such as caring for the grandchildren or elderly relative[43], and the need to “plan walking activity to avoid hills and allow greater time”[41]. Participants in two studies however, were not specific and stated generally perceived time constraints[39,45].

Other personal barriers: Other barriers reported included not having many symptoms generally or following invasive therapy[38], lower socioeconomic status[42,45], “feeling of isolation, and dependence, missing previous life and conforming to restricted life”[46].

#### Walking activity related barriers

Walking induced pain: Walking-related claudication pain limitation was the most frequently identified behavioral barrier. Ten studies representing the only mixed method design[38], all (100%) of the qualitative[38–41,46], and four (33%) of the quantitative studies[42,45,53,55] reported pain related to walking as a barrier. Participants in the qualitative and mixed method studies commonly cited leg pain, discomfort or the need for frequent stops due to leg pain as a barrier to engaging in walking exercise or walking to a therapeutic intensity. Barbosa et al[45], a quantitative study, investigated the prevalence and predictors of barriers to physical activity, and showed that exercise-induced pain was the most frequently reported personal barrier. Also the need to rest because of pain predicted lower physical activity[45]. Another quantitative study[42], which investigated the relationship between barriers to physical activity and the sociodemographic and clinical characteristics of the participants identified needing to rest due to pain as a barrier. The third quantitative study investigated mediators of walking exercise and reported that people who experience bilateral claudication pain had weaker intention to walk than those with unilateral pain[53]. The fourth quantitative study[55] investigated barriers to PA and reported walking limiting pain as the most important barriers among participants.

Fatigue: Two quantitative studies reported fatigue (also described as “lack of physical energy”) as a significantly prevalent barrier[42,45] that was associated with declining physical activity[45] among participants. Similarly, participants in one qualitative study[39] indicated that they do not engage in walking exercise due to fatigue and low energy levels.

Lack of motivation: Lack of self-motivation to exercise (also described as “lack of confidence” “lack of conscientiousness” or “lack of interest” by researchers) was another behavioral barrier frequently identified among participants in qualitative[39–41], and mixed method studies[38]. Also, one quantitative study[54] showed that lack of interest in exercise was the second most frequent reason given by participants for non-attendance in a community-based SEP.

No walking advice or lack of specific walking advice: Two qualitative studies[40,41] and one mixed method study[38] reported barriers related to receiving advice; two major themes emerged from these studies. The first theme was that participants did not engage in walking exercise because they did not receive instruction to walk from the health professionals[38]. The second theme was that the instructions given by the health professionals were neither specific, tailored, purposeful nor accompanied with any walking plan[38,40,41]. Interestingly, lack of advice or lack of specific advice as a barrier to walking exercise was not examined as a potential barrier in any of the included quantitative studies.

Lack of supervision: One mixed method study noted lack of supervision during walking exercise was a barrier to continuing in walking exercise[38]. Lack of monitoring during physical activity was reported as a prevalent barrier in one of the quantitative studies[45]. No qualitative study investigated lack of supervision as a barrier to engaging in walking exercise/physical activity.

#### Environment related barriers

Adverse weather conditions: Inclement weather was the most frequently identified environmental barrier. Unfavorable weather/season (e.g. winter) emerged as a barrier to walking exercise among participants in two qualitative[39,41] and one mixed method studies[38]. Also two quantitative studies[42,45] reported unfavorable weather as a barrier to participants engagement in physical activity.

Lack of/poor walking surface/obstacles that worsen pain: Absence of, uneven or poorly maintained pavements, or the presence of slopes or stairs were cited as barriers to walking[41,42,45,46]. Also, the presence of obstacles that exacerbated leg pain and not having a place to rest/sit when experiencing leg pain were reported as barriers associated with lower physical activity[42,45,55].

Lack of green areas/neighborhood/local facilities to engage in physical activity: Two quantitative studies reported lack of green areas, or not having facilities including SEP centers as a barrier to engage in walking exercise[42,45]. Similarly, participants in one study indicated security concerns and concern posed by vehicle movements as barrier to engaging in physical activity[45].

### Enablers to walking exercise

**Fig 3:**
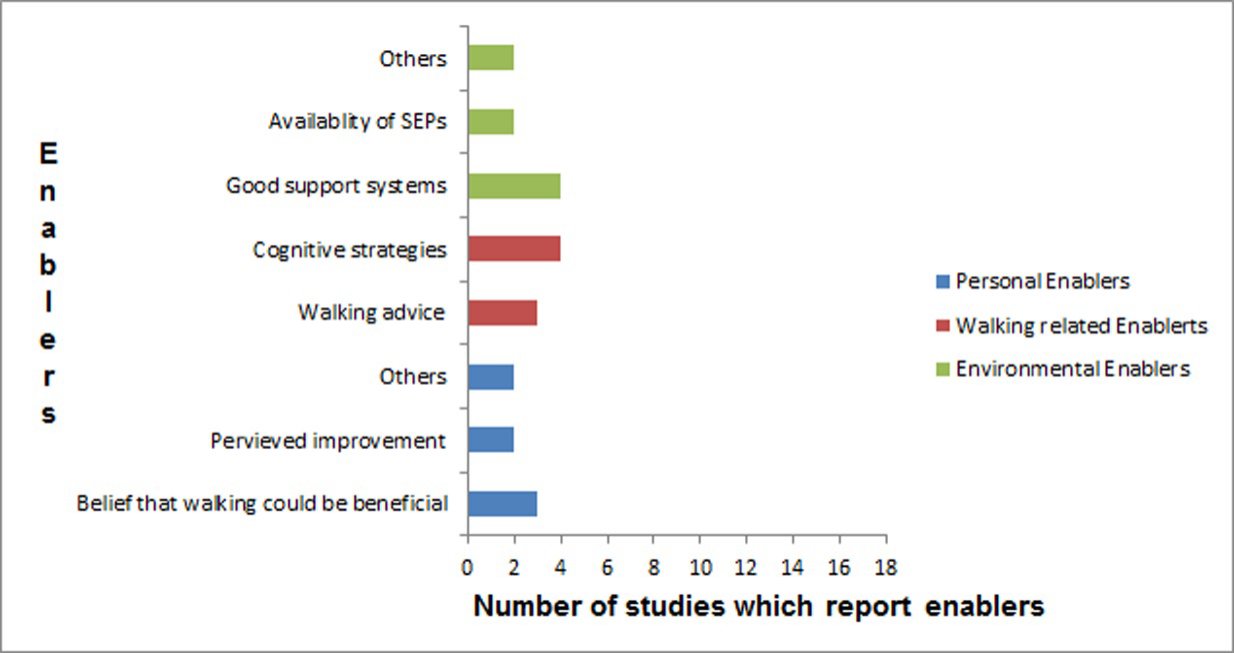
Enablers to walking in individuals with intermittent claudication and number of studies which reported them.

Enablers to walking exercises and the number of studies which reported on them are presented in Fig 3.

#### Person related motivators

Belief that walking exercise could be beneficial: Belief about the potential benefit of walking was reported as enabler in two qualitative studies[40,41], and one mixed method[38] study. Participants indicated that their belief that walking could improve, slow down the deterioration of symptoms, and or potentially replace higher risk interventions, motivated them to engage in walking exercise.

Perceived improvement: Two themes related to participants’ perceived improvement as a motivator emerged from one qualitative[41] and one mixed method[38] study. The first theme was that participants belief in potential improvement or perceived improvement in general health motivated them to continue walking exercise[38]. The second was that participants’ perceived improvement in claudication symptom motivated them to continue walking exercise[38,41].

Other motivators: Other personal motivators identified include good understanding about IC, family history of serious vascular disease like amputation acting as warning[38], history of ischemic heart disease or hypercholesterolemia[54], and good baseline maximal walking distance[52].

#### Walking behavior related motivators

Walking advice: Themes related to walking-related advice as a motivator to walking were reported in three studies. Receiving advice was the most important determinant of patients undertaking walking exercise[38]. Further, participants indicated that their desire to follow the walking advice provided by the health professionals motivated them to engage in walking exercise[38]. Also, participants in two qualitative studies indicated that a specific, purposeful, and tailored instruction about walking was a motivator to them to engage in walking[40,41].

Using cognitive strategies: Cognitive strategies as a motivator was identified in three qualitative studies and three subthemes were described. The first subtheme was related to planning walking into daily life[40]. The second subtheme was that objective walking goal setting enabled participants to actually undertake walking [46]. The third subtheme was that participants were able to continue to walk in spite of pain using mental (e.g. positive self-talks) and behavioral (e.g. stopping to take breaks) pain-coping strategies[39]. Similar to the cognitive strategies, two studies reported that participants’ perceived behavioral control[53], and having the intention to walk[39] were strong motivators for them undertaking walking.

#### Environmental related motivators

Support system: Two qualitative and one mixed method study reported three subthemes describing support systems as a motivator to engaging in walking exercise. The first subtheme was the social and emotional support, including companionship, provided by family members and animal pets[38,39,46]. The second subtheme was the group members’ support systems (derived from e.g. a group exercise program or patient support groups)[38,39,46]. The third subtheme was the encouragement and support given by the health and social care professionals[39,40,46].

Availability of SEP: Availability of supervised exercise program was identified as a motivator to engaging in walking in two qualitative studies[39,40].

Others: Other environment related motivators identified include supervision and/or some form of monitoring[40], and having place to rest when having leg pain[46]

## Discussion

This integrative review allowed the use of a social cognitive theory framework to categorize literature about barriers and enablers to walking activities in individuals with PAD and IC. It also provides a conceptual framework to develop a self-management program to enhance uptake and adherence to walking in this population. A broad range of personal, walking activity related and environmental barriers and enablers that influence walking exercise were identified. Although the majority of included studies were descriptive and qualitative studies, the study designs were appropriate for the review objective which focused on the identification of barriers and enablers rather than on the impact of a specific intervention or the evaluation of impact.

Personal (as opposed to environmental) factors were the mostly frequently explored barriers in the included studies. At the personal level, review findings highlighted comorbid health concerns, low walking capacity, and lack of knowledge (e.g. disease understanding) as the most common barriers to walking. Walking limiting pain or fatigue symptoms and lack of motivation were the most frequently reported walking activity related barriers. Similarly, the most frequently identified environmental barriers were adverse weather, and poor walking surfaces presenting obstacles that worsen pain. In contrast, benefits from cognitive strategies (such as prior planning of walking, goal setting and behavioral pain-coping strategies), and support systems (such as family, patient group, and healthcare environment that provided social, emotion and information support) were the most reported walking related and environmental enablers, respectively. Also, the perception of walking as either having potential or actual benefit was the most common personal enabler for individuals to engage or continue with walking.

The identified barriers and enablers in this review give insight into important considerations for planning walking interventions, and also provide information on specific factors that may assist in overcoming anticipated barriers. First, individuals with IC present with high prevalence of comorbidities, reduced walking capacity, and typically lack understanding of their disease pathology, risk factors and the benefit of walking[53,54]. Although several comorbid conditions may prevent individuals with IC from participating in a standard SEP, exercise is essential to reduce the impact of many of these comorbidities (e.g. hypertension, diabetes, advanced age, obesity, metabolic syndrome), reported as barriers in the included studies[38,40,43,46,58–60]. With some tailoring around specific walking advice, individuals’ specific characteristics, including barriers due to concerns about comorbidities may be addressed. An important aspect of tailoring might be starting on a lower walking regime commensurate to their walking capacity. For instance, as a preparation to the recommended 3x 30–60 minutes of walking exercise beyond the point of pain, advice may be tailored to patients to undertaking more frequent walks up to the point of exhaustion or the onset of pain, whichever comes first. This way exercise dose will be gradually increased as a progression towards the recommended level. Also, individuals should be educated on the benefits of walking exercise as a component of risk factor management beyond IC symptom improvement. Indeed, an individual’s perception of walking as having potential or actual benefit from walking is the most common personal enabler to engage in or continue walking in spite of pain. Individuals who opt to undergo a home-based exercise program should also receive the education and should be supported with some form of supervision as encouragement to engage in walking. The use of exercise diary, follow up calls, goal-setting and self-feedback using a pedometer are some of the ways to do this within a home-based exercise program.

In addition to the personal barriers, walking limiting pain or fatigue is characteristically unique to individuals with IC. Individuals with IC only experience pain after they have been walking, and which gets better with rest. This suggests that an approach to pain management which considers both the intermittent nature of the pain and fatigue, and its association with walking (the behavior of interest being prevented) may be a uniquely important consideration. For this reason, previous systematic reviews have explored alternative strategies towards pain management during exercise, for example, recommending exercising more frequently at threshold of mild pain[61], or the need to investigate alternative forms and prescriptions of exercise other than walking (e.g. polestriding, leg and arm ergometry, and resistance exercise[62,63]. In addition to those recommendations, pain barriers could potentially be addressed by a suitably structured walking program, pain management and patient education based on behavioral change techniques (cognitive strategies). By deferring the onset of pain and providing motivation to walk through structured patient education, individuals may be able reach the recommended walking guidance for therapeutic benefit.

Both physical and social environmental factors were identified as impacting on walking in individuals with IC. Again, while many of these environmental factors are common to the general population, some were unique to individuals with IC. For instance while the availability and accessibility of SEPs to individuals with IC may encourage more individuals to participate[39,40], removing physical barriers, like lack of green area, security concerns, lack of/poor pavements, and lack of local facility for walking may further encourage them to translate this to walking in the community. Some other factors such as uneven or poor walking surface, and stairs might present an unequal demand on the lower limb muscle of individuals with IC, making pain come on more quickly. Similarly, the reluctance to engage in walking due to fear of not having a place to rest when experiencing leg pain may be greater in individuals with IC. Beyond the policy implications for the physical environment, these findings highlight the importance of considering how walking programs for these individuals may be tailored to overcome these barriers.

Other environmental factors, such as the social environment, gave credence to the role of the family, healthcare and social support systems in influencing walking exercise in this population. Specifically, IC individuals emphasized benefit of having a walking partner[38,39,46], encouragement from health professionals[39,40,46], and the value of patient support groups[38,39,46]. Whether it is a SEP, home-based exercise walking program, or alternative exercise program, the potential benefits that these support systems may provide should be harnessed to enhance individuals’ engagement and adherence to the exercise.

### Implication for designing walking exercise interventions for individuals with IC

Walking limitations in individuals with IC impact physical function, social participation, quality of life and overall disease outcome[1,2] [4] [3]. Inactivity in this population accelerates disease progression and accentuates the risk of cardiovascular events[4] [3]. The findings from this review highlights the factors and constructs, within a social cognitive framework, enabling a comprehensive understanding of what makes patients’ engagement with, and adherence to, walking difficult. This has important implications. Personal, behavioral and environmental barriers may need to be addressed to promote and sustain adoption of walking exercise, either in SEPs or home-based exercise programs. Addressing comorbid health issues with patient education and appropriate exercise tailoring may benefit a larger proportion of patients, who often have comorbid health conditions. Similarly, a pain management strategy that prolongs time before pain becomes unbearable may, not only encourage patients to engage in walking, but may also prolong the time to reach pain tolerance, enabling them gain the maximum therapeutic benefit from walking. Finally, with a good support system (provided by health professionals, patient groups and relatives) and an intervention tailored with the understanding of patient physical environments, it is possible that patients will be encouraged to form healthy exercise habits. One possible consideration is how structured education, delivered alongside patient-centered pain management with or without SEP, may be used to boost walking exercise engagement and adherence in individuals with PAD and IC. The MRC guideline for complex intervention development[64] provides a framework for the development of such interventions. An important next step to developing such an intervention would be the identification of the useful components of pain management and patient education, which may need to be accomplished through systematic literature reviews.

### Limitations

This review has several limitations. First, it was based only on English language peer review literature of published research. Other relevant studies published in different languages may be available. Secondly, it is acknowledged that the demographic and disease characteristics of participants varied across included studies, potentially impacting on what influenced walking behavior. To account for these variations, attempts were made to identify factors within, but not limited to, the framework. Nevertheless, other than identifying patients’ characteristics as a barrier and/or enabler, conclusions specific to these characteristics within the heterogeneous group of patients with IC could not be drawn. Thirdly, the majority of the included studies were qualitative and non-experimental designs, therefore, identification of causal factors responsible for walking (or not walking) was lacking. In addition, only two of the included studies implemented randomization in sample group allocation, limiting generalization to sample populations. However, the type and quality of studies included in this review were considered adequate and fit the review objective which was to identify factors, rather than establishing causal and effect relationship these factors or of an intervention. Finally, another limitation of this review is the preliminary nature of the proposed framework within this topic. However, a relatively large number of studies were included, and given that this is the first review on factors influencing walking specific to individuals with IC from PAD the findings contribute to a comprehensive and deeper understanding of walking exercise in this patient population.

### Conclusion

The common barriers to walking exercise among patients with IC are comorbid health concerns, walking limiting pain, and lack of motivation. Patients’ poor understanding of the disease and lack of clear walking advice encourages the belief that walking is harmful. Patients need encouragement, support and feedback to engage and adhere to walking exercise recommendations. The review findings indicate that patients have different barriers and enablers indicating that a one-size-fits-all walking exercise programs may not be a solution for all eligible patients with IC. Therefore, practice needs to adopt a patient-centered approach, particularly addressing disease understanding, via patient education, and walking limiting pain to overcome barriers and increase walking exercise engagement and adherence among patients with IC. Similarly, future research that explores the useful components of pain management and patient education interventions and how these can be used to develop targeted patient-centered walking interventions in patients with IC is essential.

## Supporting information

S1 Fig A search strategy implemented in CINAHL. (TIF)
