# Supplementary figures and images for: Barriers and enablers to walking in individuals with intermittent claudication: A systematic review to conceptualize a relevant and patient-centered program
Source: PLoS One. 2018 Jul 26;13(7):e0201095. doi: 10.1371/journal.pone.0201095 (PMC6062088; doi:10.1371/journal.pone.0201095)

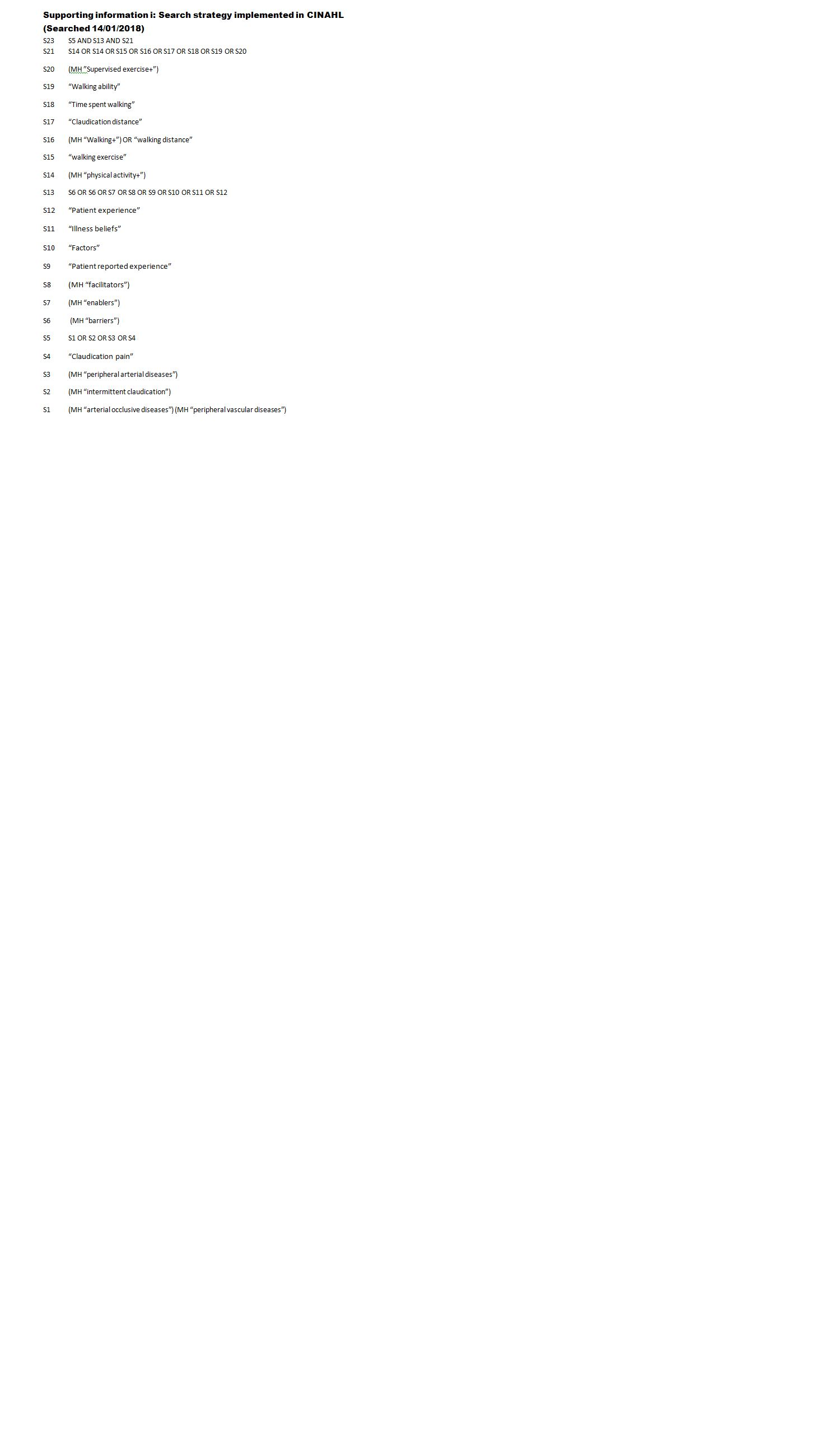

Supplement: S1 Fig — (TIF) [file pone.0201095.s001.tif]
